# Advances in Research on Isothermal Signal Amplification Mediated MicroRNA Detection of Clinical Samples: Application to Disease Diagnosis

**DOI:** 10.3390/bios15060395

**Published:** 2025-06-18

**Authors:** Yu Han, Xin Sun, Sheng Cai

**Affiliations:** 1 School of Pharmaceutical Sciences, Jilin Medical University, Jilin 132013, China; hanyu.jlmu@vip.163.com; 2 Zhejiang Province Key Laboratory of Anti-Cancer Drug Research, College of Pharmaceutical Sciences, Zhejiang University, Hangzhou 310058, China

**Keywords:** miRNA detection, isothermal signal amplification, point-of-care testing, disease diagnosis

## Abstract

With the rapid development of modern molecular biology, microRNA (miRNA) has been demonstrated to be closely associated with the occurrence and development of tumors and holds significant promise as a biomarker for the early detection, diagnosis, and treatment of cancer and other diseases. Therefore, detecting miRNA and analyzing it to determine its biological functions are of great significance for the screening and diagnosis of diseases. However, the intrinsic characteristics of miRNAs, including their low abundance, short sequence lengths, and high family-specific sequence homology, render traditional detection methods such as Northern blot hybridization, microarray use, and reverse transcription quantitative PCR (RT-qPCR) inadequate for meeting the stringent requirements of clinical detection in biological samples, a task requiring accuracy, rapidity, high detection power, specificity, and cost-effectiveness. In recent years, a substantial amount of effort has been put into developing innovative methodologies to address these challenges. In this review, we aim to provide a comprehensive overview of the recent advancements in these methodologies and their applications in clinical biological sample detection for disease diagnosis.

## 1. Introduction

MicroRNAs (miRNAs) are a class of non-coding RNA (ncRNA) molecules approximately 22 nucleotides in length that play critical roles in biological processes such as cell differentiation, proliferation, apoptosis, and metabolism by regulating gene expression [1,2]. Since the discovery of the first miRNA (lin-4) in Caenorhabditis elegans in 1993, thousands of miRNAs have been identified, many of which are closely associated with the pathogenesis of cancers, cardiovascular diseases, neurodegenerative disorders, and infectious diseases [3,4]. Thus, miRNA’s discovery has significant implications for the study of gene functions, the prevention and treatment of human diseases, and the exploration of biological evolution.

MiRNA is abundant in tissues as well as biological fluids [5]. Therefore, it has become a good biomarker for various diseases. To date, nearly 4000 human miRNAs have been identified, with at least 30% demonstrating potential as novel diagnostic indicators for human pathologies [6]. Crucially, these aberrant miRNA expression profiles not only signal early-stage disease onset but also reflect disease progression, metastatic potential, therapeutic responses, and drug resistance mechanisms. Consequently, miRNAs have been exploited as powerful analytical tools for cancer diagnosis, prognostic stratification, and precision treatment prediction.

Due to its specific expression characteristics in tumor tissues and cells and the metabolic effects of blood circulation, miRNA can serve as a non-invasive and valuable cancer biomarker in biological fluids (blood, urine, and saliva). As reported in the literature [7,8], the levels of miR-221, miR-222, miR-146b, and miR-155 in the plasma of papillary thyroid carcinoma (PTC) patients show an upward trend in comparison with those in the plasma of patients with benign lesions and healthy individuals. Moreover, miR-222, miR-146b, and miR-155 can distinguish PTC from benign lesions, as the miRNA in tumor tissues may be released into the blood and then transported through the circulatory system to exert its biological functions. Therefore, miRNA in the blood can serve as a potential biomarker and analytical tool for non-invasive cancer diagnosis, and blood sample acquisition has better patient compliance than fine-needle aspiration biopsy (FNAB). Table 1 summarizes the potential of miRNA in diagnosing cancer in different biological fluids in recent years [9,10,11,12,13,14,15,16,17]. Although miRNA has been detected in various biological fluids and applied to disease diagnosis, since it comes from different sources and exists in different forms in biological fluids, its content is relatively low, making it difficult to detect using traditional methods. Moreover, the short sequence length, low expression levels, and high similarity among homologous family members of miRNAs pose significant challenges for their precise detection. Therefore, developing miRNA analysis methods with high detection power and specificity is of great significance for life sciences and pathology research.

In recent years, because of the convergence of molecular biology, nanotechnology, and artificial intelligence, miRNA detection technologies have undergone transformative advancements, evolving from traditional qualitative analysis to highly sensitive quantitative detection and from single-marker screening to multi-omics integration. This article systematically reviews the developmental progress of miRNA detection technologies and their applications in clinical diagnosis, basic research, and point-of-care testing (POCT) and explores potential directions for future technological optimization and clinical translation.

## 2. Limitations of Traditional Detection Methods and the Need for Technological Advancements

In the early stages, miRNA detection primarily depended on Northern blotting and reverse transcription quantitative PCR (RT-qPCR). Northern blotting involves the use of electrophoresis to separate RNA, followed by probe hybridization. While capable of confirming the molecular weight of miRNA, this method suffers from a poor detection limit, labor-intensive protocols, and high sample requirements, rendering it impractical for clinical applications [18]. RT-qPCR, utilizing stem–loop primers or poly-A-tailing strategies to amplify miRNAs, can achieve significantly enhanced sensitivity (down to the fM level). However, its reliance on intricate primer design and thermal cyclers restricts its utility in resource-limited settings [19].

Meanwhile, high-throughput sequencing technologies like RNA sequencing (RNA-seq) enable genome-wide miRNA discovery and expression profiling. Yet, their prohibitive costs, requirement for computationally intensive data analysis, and dependence on bioinformatics expertise confine their use largely to research rather than clinical practice [20].

The constraints of these conventional methods have driven the exploration of more efficient, portable, and cost-effective alternatives. Notably, isothermal amplification techniques—which eliminate the need for complex instruments and their integration with advanced interdisciplinary technologies (e.g., nanomaterials, microfluidics, and biosensors)—have emerged as focal points in recent methodological innovations [21,22,23].

## 3. The Rise in Isothermal Nucleic Acid Amplification Technology and Innovative Strategies for Its Use

Isothermal nucleic acid amplification enables the efficient amplification of nucleic acids at a constant temperature. Unlike traditional PCR, is does not require thermal cycling, significantly reducing equipment requirements and operational complexity. In the past five years, miRNA detection techniques based on isothermal amplification have achieved dual breakthroughs in terms of detection limit and specificity through enzymatic optimization, innovative probe design, and the combination of multiple technologies. This section focuses on the integration of mainstream isothermal amplification techniques with advanced signal transduction strategies, systematically examining their current applications and technical challenges in detecting miRNA in complex biological samples. The isothermal amplification techniques that have been widely utilized in recent years include rolling circle amplification (RCA), duplex-specific nuclease signal amplification (DSNSA), catalytic hairpin assembly (CHA), strand-displacement amplification (SDA), hybridization chain reaction (HCR), loop-mediated isothermal amplification (LAMP), and exponential amplification reaction (EXPAR), all of which are described in the following section.

### 3.1. Rolling Circle Amplification (RCA)

**Figure 1:**
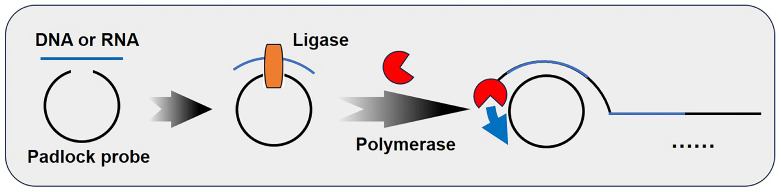
Schematic diagram of miRNA detection methods based on rolling circle amplification (RCA). This material was reprinted (adapted) with permission from Ref. [19]. Copyright 2017 American Chemical Society.

Figure 1 demonstrates the basic principle of the reaction. The target nucleic acid molecule (DNA or RNA) undergoes specific hybridization with the terminal bases of a linear DNA template (a padlock probe). At this stage, the 3′ hydroxyl end (3′-OH) and the 5′ phosphate end (5′-PO_4_) of the DNA probe are brought into proximity, enabling their ligation into a circular structure via T4 DNA ligase. Subsequently, under the action of DNA polymerases such as Phi29 DNA polymerase and deoxyribonucleoside triphosphates (dNTPs) (including deoxyadenosine triphosphate (dATP), deoxycytidine triphosphate (dCTP), deoxyguanosine triphosphate trisodium salt (dGTP), and deoxythymidine triphosphate (dTTP)), the target nucleic acid molecule is amplified linearly according to the template. The reaction products can be detected using techniques such as gel electrophoresis, fluorescent nucleic acid dyes, colorimetric assays, and electrochemical biosensors [24].

In addition to the single-primer RCA method, there are four other approaches: hyperbranched RCA, multi-primers RCA, padlock probe RCA, and netlike RCA [25]. Simply put, the multi-primer method involves designing multiple single-stranded primers complementary to the sequence of the circular template DNA probe, which is continuously displaced by DNA polymerase during the RCA reaction, thereby enhancing the efficiency of product accumulation. Hyperbranched RCA introduces multiple primers that not only bind to and extend from the linear RCA products but also generate DNA products capable of interacting with other primers, enabling exponential amplification. Padlock probe RCA makes use of linear oligonucleotides. Once these oligonucleotides bind to specific targets, they circularize and function as scaffolds for RCA, endowing genotyping, mutational analyses, and the detection of nucleic acid markers with an extremely high level of specificity. Netlike RCA forms a complex network-like structure through primer extension on circular templates. The endonuclease-mediated generation of trigger strands is exploited and subsequently integrated with other signal output modalities.

Kuhn et al. reported that T4 DNA ligase can ligate single-stranded DNA (ssDNA) ends even in the absence of a double-stranded template, leading to high background signals and false-positive results [26]. In contrast, T4 RNA ligase strictly reacts with double-stranded RNA or DNA-RNA hybrid templates, providing a prerequisite and foundation for using RCA to detect miRNAs.

Various strategies based on RCA for miRNA detection have emerged in recent years, offering new insights into high-detection-power and highly specific miRNA detection methods. For instance, Jiang et al. developed a colorimetric detection method for identifying miR-143 using gold nanoparticles (AuNPs) combined with CRISPR-Cas12a and exponential RCA [27]. This approach enabled the visualization of miR-143 at a concentration as low as 1 fM through colorimetric assays and the detection of analytes at aM levels via UV–Vis spectroscopy. While the integration of RCA with other methods enhances detection limits in miRNA detection, the inherent limitations of RCA require urgent resolution. (1) The requirement for a two-step reaction: RCA detection involves two sequential reactions and requires two functional enzymes (e.g., ligase and polymerase) along with phosphorylated DNA probes, resulting in elevated detection costs and prolonged reaction times. (2) Template-dependent limitations: Since RCA heavily relies on the binding of DNA templates and initiators, impurities in unpurified precursor miRNA samples from cellular extracts may also bind to the template, leading to false-positive results. These challenges underscore the need for further optimization of RCA-based miRNA detection methods to enhance their practicality and reliability in clinical applications.

### 3.2. Duplex-Specific Nuclease-Assisted Signal Amplification (DSNSA)

**Figure 2:**
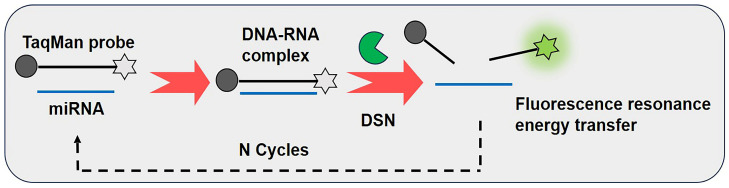
Schematic diagram of miRNA detection methods based on duplex-specific nuclease signal amplification (DSNSA). The black circle represents the quencher group, the gray pentagram represents the quenched fluorophore, and the green glowing pentagram represents the fluorophore with restored fluorescence. This material was reprinted (adapted) with permission from Ref. [19]. Copyright 2017 American Chemical Society.

As shown in Figure 2, the DSNSA reaction requires a dual-modified DNA probe (a quencher group and fluorophore), whose base sequence is complementary to that of the target miRNA [28]. The TaqMan fluorescent probe specifically binds to the target miRNA. Under the action of the DSN enzyme, the DNA strand in the double-stranded hybrid complex is hydrolyzed, causing the quencher group to move away from the fluorophore. Based on the principle of fluorescence resonance energy transfer (FRET), the fluorescent signal is restored. Moreover, the miRNA maintains the integrity of its bases during the reaction process. Therefore, it can bind to unreacted TaqMan fluorescent probes multiple times, forming n cycles to achieve signal amplification. This method can achieve a relatively wide dynamic detection range (100 fM–100 nM), and its limit of detection (LOD) is at least five orders of magnitude lower than that of the traditional molecular beacon (MB)-based method [29]. Based on the aforementioned fluorescent probe and DSN enzyme, Shen et al. established a detection method in which magnetic beads were coupled with fluorescent nucleic acid probes to bind to the target miRNA, reducing the detection limit to 60 fM and applying this method to the detection of total cellular RNA [30].

In recent years, the strategy of combining the DSNSA method with high-performance liquid chromatography coupled with a fluorescence detector (HPCL-FLD) [31], tandem mass spectrometry (MS/MS) [32], and inductively coupled plasma–mass spectrometry (ICP-MS) [33] for miRNA detection has been widely used. Usually, the fluorescent molecule modified on the DNA probe that binds to the target miRNA is replaced with mass tags (small molecules or polypeptide chains) that can be highly sensitively detected by the detector used. Therefore, through the labeling of mass tags or fluorescent groups on DNA probes in conjunction with the analytical capabilities of chromatography and MS, it is possible to simultaneously analyze and quantify multiple miRNAs. These strategies can detect miRNA at fmol levels or even lower concentrations and have been applied in complex biological matrices, demonstrating that the DSN enzyme is an effective tool for multiple-miRNA detection.

Although this isothermal analysis strategy reduces the use of enzymes in comparison with the RCA method and can detect the target analyte in a one-step reaction, it also has non-negligible drawbacks: (1) Since the design of the DNA probe depends on the base sequence of the target miRNA, when detecting different miRNAs, the melting temperature (Tm) of the DNA probe used also changes, resulting in a change in the optimal hybridization temperature between the miRNA and the DNA probe. Therefore, the reaction conditions need to be re-optimized. (2) The DSN enzyme relies on Mg^2+^ to function. When combined with other functional enzymes or other isothermal amplification methods, it is difficult to unify the reaction components. (3) Most of the DNA probes using the DSNSA method need to be labeled with at least one detection group (a fluorescent molecule or polypeptide chain), resulting in a relatively high detection cost.

### 3.3. Catalytic Hairpin Assembly (CHA)

**Figure 3:**
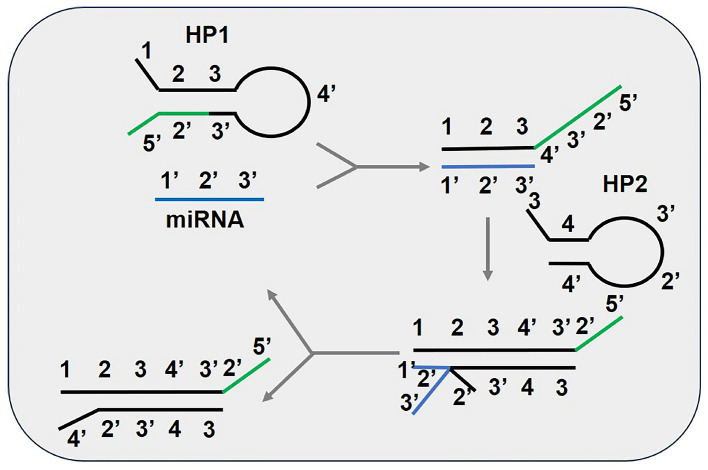
Schematic diagram of miRNA detection methods based on catalytic hairpin assembly (CHA). This material was reprinted (adapted) with permission from Ref. [19]. Copyright 2017 American Chemical Society.

As shown in Figure 3, the CHA reaction requires two hairpin primers (HPs), denoted as HP1 and HP2. Each region of these primers is represented by numbers, with each region containing 6–12 base sequences. The symbol “ ′ ” represents the complementary base sequence corresponding to the numbered regions. Initially, both HP1 and HP2 exist in a closed state in solution, maintaining stability. Upon the introduction of a catalytic chain (C1) or a target DNA/RNA molecule into the reaction, C1 binds to the sticky-end region of HP1 via complementary base pairing, forming a C1-HP1 duplex assembly. Subsequently, region 4 of HP1 becomes exposed and binds to the sticky-end region of HP2, initiating a strand migration process. This process results in the replacement of C1 by HP2, ultimately forming the final product, an HP1-HP2 duplex assembly. Once C1 is released back into the solution, it can rebind to other unreacted HP1 molecules, repeating the cycle and continuously generating new HP1-HP2 duplex assemblies [34].

In addition to the dual-hairpin CHA reaction, studies have reported CHA reactions involving three, four, or even more hairpin primers [35,36]. This isothermal linear amplification reaction overcomes the limitations of protease-mediated signal amplification, such as complex operational and reaction conditions and high costs. As a result, CHA has become a robust platform for constructing enzyme-free amplification-based biosensors with high detection power for nucleic acids, proteins, and small molecules. Furthermore, CHA has been successfully applied to in situ or cellular imaging [37], offering new perspectives for disease diagnosis and treatment.

To confirm the formation of CHA and quantify its products, researchers recently integrated this reaction with various detection techniques, including fluorescence labeling [38], nanomaterials [39], DNAzyme-assisted signal amplification [40], strip-based colorimetric assays [41], chemiluminescence [42], and electrochemical methods [43]. These integrations have significantly enhanced the sensitivity of miRNA detection. Our group recently focused on integrating the DSN enzyme with CHA and DNA walker to develop miRNA detection methods based on fluorescence and chemiluminescence [44,45,46]. The signal amplification capability of DSN significantly enhanced the detection performance of CHA.

Despite its advantages, including operational simplicity, high sensitivity, and strong specificity, detailed studies describing the design principles of hairpin primers for CHA are scarce. Additionally, challenges such as improving reaction efficiency and minimizing background leakage must be addressed to fully harness the potential of CHA in diagnostic applications. Addressing these issues will be critical for advancing CHA-based detection methods toward practical clinical applications.

### 3.4. Strand-Displacement Amplification (SDA)

**Figure 4:**
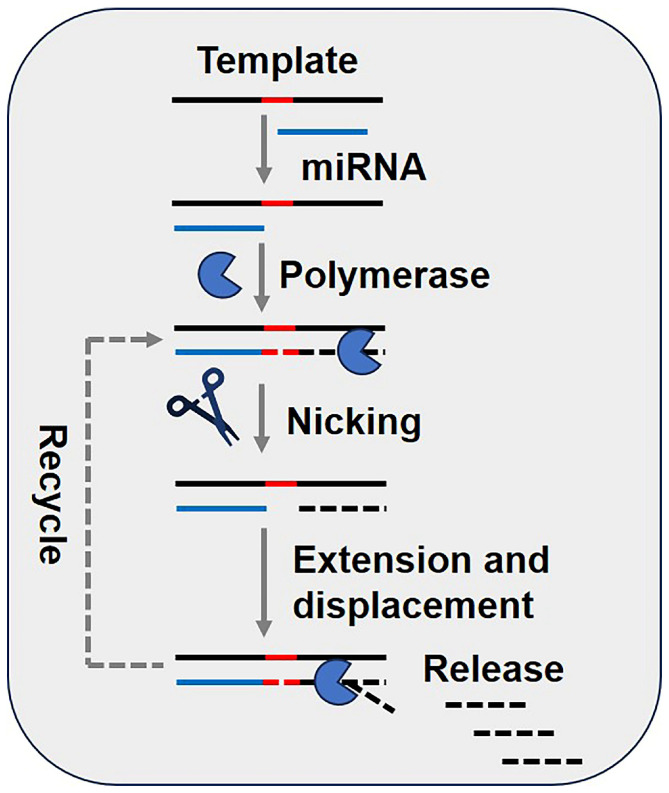
Schematic diagram of miRNA detection methods based on strand-displacement amplification (SDA). The red dashed line represents the recognition site of the nicking endonuclease. This material was reprinted (adapted) with permission from Ref. [19]. Copyright 2017 American Chemical Society.

As illustrated in Figure 4, the SDA reaction primarily involves a restriction endonuclease and a DNA polymerase with strand displacement functionality. The process begins with the hybridization of the DNA template to the miRNA, followed by extension. The restriction endonuclease recognizes specific base sequences and introduces a nick in the double-stranded DNA (dsDNA). The polymerase then extends along the nicked region from the 3′ end towards the 5′ end, displacing the 5′ end sequence. This extension and displacement process iteratively repeats, accumulating numerous ssDNA products and achieving signal amplification [47].

The SDA reaction has undergone rapid development in the field of miRNA detection due to its rapidity, efficiency, suitability for isothermal conditions, etc. Li et al. [48] established an innovative fluorescent biosensor for detecting miR-21 by integrating hyperbranched RCA with SDA. This system demonstrated high detection power with an LOD of 16 fM. Through the meticulous design of the RCA template, reverse primer, HP1, and HP2, these researchers successfully avoided the generation of false-positive signals, a common issue in traditional methods. Additionally, this system is versatile and can be employed for the detection of metal ions, proteins, and other biomolecules, offering effective tools and new perspectives for environmental monitoring and clinical diagnostics.

Despite its potential, the SDA technique is not without limitations. (1) Complex primer design: The design of primers for SDA is intricate, leading to inconsistent product uniformity and the generation of byproducts that interfere with detection. (2) Restrictions on polymerase selection: The choice of DNA polymerase is limited, as it must lack 5′−3′ exonuclease activity. While SDA currently has some shortcomings, its integration with other amplification methods presents promising avenues for disease diagnosis and research, particularly as nucleic acid testing continues to advance rapidly.

### 3.5. Hybridization Chain Reaction (HCR)

**Figure 5:**
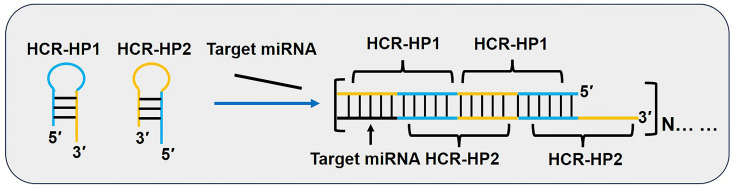
Schematic diagram of miRNA detection methods based on hybridization chain reaction (HCR). In primers HP1 and HP2, identical colors denote complementary base sequences. The miRNA indicated by the black line is capable of recognizing the yellow region in primer HP1. This material was reprinted (adapted) with permission from Ref. [19]. Copyright 2017 American Chemical Society.

As shown in Figure 5, as in the CHA reaction, HCR requires two hairpin primers (HP1 and HP2). In the absence of a trigger strand (DNA or RNA), both primers remain stable and self-contained. Upon introducing the trigger strand, HP1’s anchor region binds to the trigger strand via complementary base pairing, destabilizing the secondary structure of HP1. This destabilization exposes the stem region of HP1, which subsequently binds to the stem region of HP2, opening its secondary structure. The stem region of HP2 then binds to the trigger strand, leading to the displacement of HP1 and restarting the cycle. This iterative process ultimately generates a nicked dsDNA copolymer [49].

The HCR technique has garnered significant attention due to its enzyme-free, isothermal, and cost-effective nature, leading to rapid advancements in signal amplification applications. Variants of HCR include visual HCR [50], fluorescent HCR [51], aptamer-based HCR [52,53], and non-linear HCR [54].

HCR has been extensively applied in miRNA detection biosensors, and, when combined with nucleic acid aptamers or antibodies, it enables in situ and live-cell imaging. These applications provide deeper insights into biological processes while also paving new avenues for disease diagnosis and therapy. Similarly to CHA, HCR is subject to certain limitations. Firstly, there is a lack of fundamental investigations into its underlying principles and the tools used to directly design primers to enhance reaction efficiency and reduce costs. Secondly, HCR is often integrated with enzyme-mediated nucleic acid amplification methods to establish high-detection-power and highly specific detection systems. However, enzymes impose stringent requirements on the reaction environment, limiting their applicability in cell imaging and complex biological sample detection. Therefore, developing enzyme-free HCR biosensors with high detection power and specificity represents a promising future research direction.

In summary, while HCR has remarkable potential in miRNA detection and therapeutic applications, addressing its current limitations and exploring innovative integration strategies will be crucial for advancing its utility in clinical diagnostics and molecular medicine.

### 3.6. Loop-Mediated Isothermal Amplification (LAMP)

**Figure 6:**
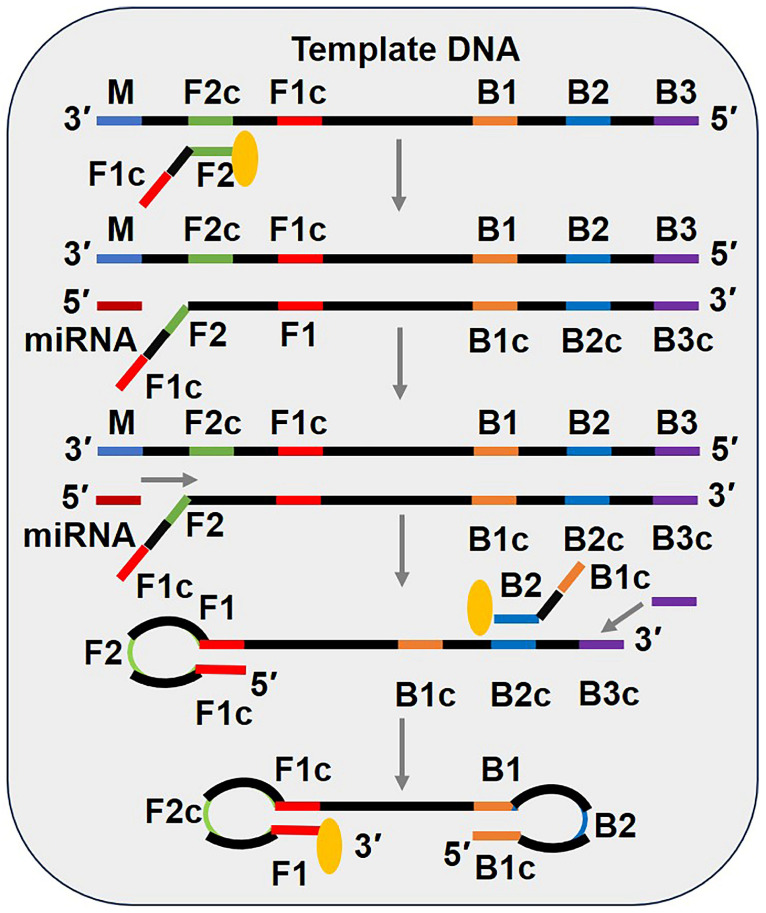
Schematic diagram of miRNA detection methods based on loop-mediated isothermal amplification (LAMP). This material was reprinted (adapted) with permission from Ref. [19]. Copyright 2017 American Chemical Society.

As shown in Figure 6, LAMP detection technology primarily involves four types of primers: template DNA, a forward inner primer (FIP), a backward inner primer (BIP), and outer primer B3. Template DNA contains six base regions: B3, B2, B1, F1c, F2c, and M. The reaction begins with the FIP binding to the F2c region of the template DNA. In the presence of a polymerase exhibiting strand displacement activity and dNTPs, extension occurs. The target analyte hybridizes with the M region, and, under enzymatic catalysis, strand displacement DNA synthesis is initiated, releasing the ssDNA connected to the FIP. Due to the complementary base pairing between F1 and F1c, a stem–loop structure forms at the 5′ end. At the 3′ end of the stem–loop ssDNA, the BIP binds to the B2c region, and extension occurs under enzymatic catalysis. Subsequently, the outer primer B3 hybridizes with the B3c region at the 3′ end of the stem–loop ssDNA, initiating strand displacement and releasing the ssDNA connected to the BIP. This product can form dumbbell-shaped DNA molecules at both the 3′ and 5′ ends. Thereafter, additional FIPs and BIPs bind to the F2c and B2c regions, respectively, of the stem–loop structure, generating numerous stem–loop structures [55].

The detection of LAMP products primarily involves methods such as precipitation assays, fluorescent dye incorporation, and agarose gel electrophoresis. These methods are widely applied in the detection of viruses, bacteria, and parasites. The LAMP reaction offers advantages such as isothermal conditions and high efficiency. Additionally, during DNA product generation, numerous byproducts are produced, resulting in white precipitates. This characteristic eliminates the need for cumbersome validation experiments, making LAMP suitable for rapid screening and detection in grassroots institutions.

In summary, LAMP is a powerful isothermal amplification technique with unique features, including the ability to generate visible precipitates and compatibility with simple detection methods. These attributes make it an ideal tool for point-of-care diagnostics and resource-limited settings.

### 3.7. Exponential Amplification Reaction (EXPAR)

**Figure 7:**
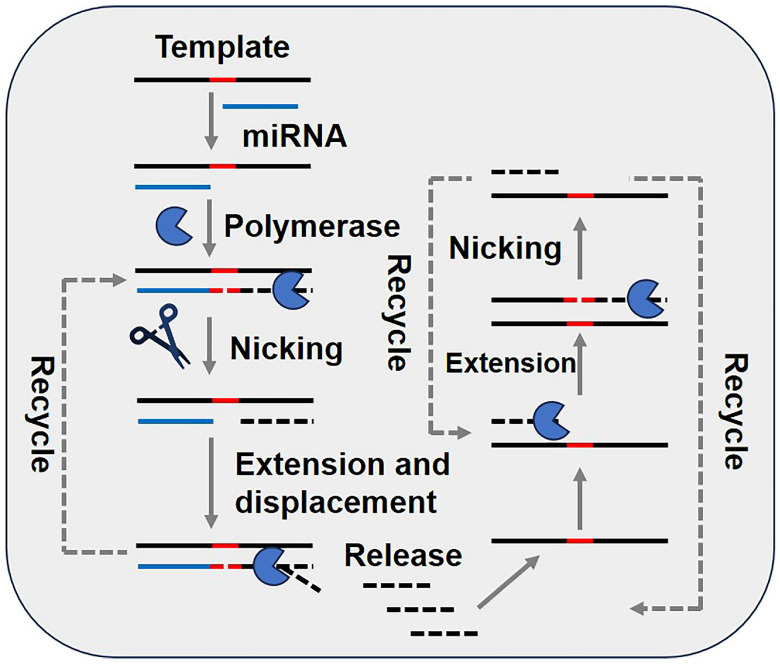
Schematic diagram of miRNA detection methods based on the exponential amplification reaction (EXPAR) technique. The red dashed line represents the recognition site of the nicking endonuclease. This material was reprinted (adapted) with permission from Ref. [19]. Copyright 2017 American Chemical Society.

As illustrated in Figure 7, EXPAR shares similarities with the SDA technique, primarily with respect to involving the use of a carefully designed DNA template, a restriction endonuclease, and a DNA polymerase with strand displacement functionality but lacking 5′−3′ exonuclease activity. The DNA template is flanked by sequences complementary to the target nucleic acid molecule, with the central region serving as the recognition site for the restriction endonuclease.

The process begins with the hybridization of the target nucleic acid molecule to the DNA amplification template. At a constant temperature and in the presence of DNA polymerase and dNTPs, extension occurs along the template in the 5′ to 3′ direction, yielding dsDNA products. The restriction endonuclease recognizes and cleaves the dsDNA at specific sites, enabling the DNA template to extend again at the nicked region. This process results in the displacement of ssDNA complementary to the target nucleic acid molecule, facilitating linear amplification of the target. The ssDNA products can hybridize with the DNA template, undergoing further exponential amplification through iterative extension, cleavage, and displacement. Ultimately, numerous dsDNA molecules are generated, which can be detected using fluorescent dyes. When detecting RNA, the released ssDNA sequence mirrors the target RNA sequence, with the ribonucleotides and uracil in RNA replaced by deoxyribonucleotides and thymine in the DNA strand [56,57].

**Figure 8:**
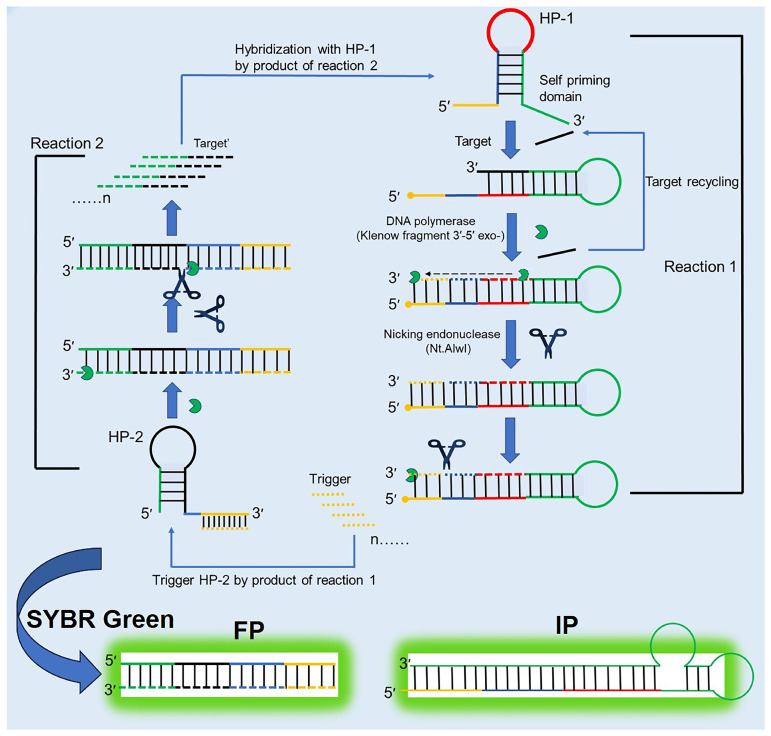
Mechanism of the SPHIA method for nucleic acid detection as depicted in a schematic diagram. This material was reprinted with permission from Ref. [58]. Copyright 2020 American Chemical Society.

However, the EXPAR technique is constrained by limitations related to the length of the target nucleic acid sequence. To address these limitations, Song et al. [58] developed the self-priming hairpin-utilized isothermal amplification (SPHIA) method. As depicted in Figure 8, HP1 consists of a 5′ complementary trigger sequence, a restriction endonuclease recognition site, a loop region for target nucleic acid recognition, and a self-priming domain at the 3′ end. In the presence of the target nucleic acid, HP1 undergoes structural changes, exposing the self-priming domain and forming a 3′ stem–loop structure. Continuous extension, displacement, and cleavage by DNA polymerase and restriction endonuclease generate abundant dsDNA intermediate products and short ssDNA fragments. The ssDNA products can trigger HP2, another hairpin primer with a 3′ restriction endonuclease recognition site, a loop region, and a stem region complementary to the target nucleic acid. Upon activation of HP2, polymerase and endonuclease collaborate to produce extensive dsDNA intermediates and ssDNA products identical to the target nucleic acid sequence. Through these interconnected reactions, a vast quantity of dsDNA is generated, enabling real-time monitoring via double-strand-specific fluorescent signals, in turn allowing for ultrasensitive detection of target nucleic acids under isothermal conditions. The SPHIA method overcomes the limitations of the EXPAR technique, enabling the detection of both long-chain DNA molecules and short-chain RNA molecules, such as miRNA. It holds significant potential for detecting target nucleic acids in real clinical samples, serving as a versatile and sensitive diagnostic tool. In summary, while the EXPAR technique provides a foundational framework for nucleic acid amplification, advancements like SPHIA expand its capabilities, addressing previous constraints and broadening its applicability in clinical diagnostics and molecular biology research.

This section primarily introduces the basic principles of and typical detection strategies for isothermal amplification techniques for miRNA detection. Individual isothermal amplification methods are insufficient to meet the demands of miRNA detection. As summarized in Table 2, which lists isothermal amplification techniques combined for miRNA detection over the past seven years (2018–2024) [31,42,59,60,61,62,63,64,65,66], several key conclusions can be drawn. (1) Electrochemical methods: While these methods offer exceptional analytical capabilities (down to aM levels), they are hindered by prolonged electrode preparation and extended reaction times. (2) Multi-enzyme and DNAzyme strategies: These approaches exhibit shorter reaction times; however, they necessitate larger quantities of templates and primers, coupled with complex primer designs. (3) HPLC-FL: Although characterized by longer reaction times, this method enables the simultaneous detection of multiple miRNAs in a single analysis without requiring wavelength adjustments or repeated injections. Additionally, it simplifies primer design and maintains a constant reaction temperature.

Therefore, the development of isothermal amplification strategies featuring simple primer designs, short reaction times, and the ability to simultaneously detect multiple miRNAs is the primary goal for future development.

## 4. Clinical Sample Detection for Disease Diagnosis

### 4.1. The Application of miRNA Detection Methods in Disease Diagnosis

It is widely recognized that the association between microRNAs (miRNAs) and the pathogenesis and progression of diseases has established their utility as versatile biomarkers for various disorders. However, their low abundance in human serum, coupled with a requirement for biological enzymes and diverse biomaterials, necessitates reaction systems typically ranging from 10 to 100 microliters. Larger reaction volumes would inherently increase the consumption of costly enzymes and materials, making the use of miRNAs impractical for routine analysis. Consequently, analyzing miRNAs in human blood with minimal reaction volumes presents a critical challenge; thus, high-detection-power methods are imperative. Traditional single-nucleic-acid isothermal amplification methods alone can no longer address these demands. Over the past three years (2023–2025), researchers have actively developed more sophisticated approaches. In Table 3, we summarize cutting-edge methods with superior sensitivity that have been successfully applied to disease detection [67,68,69,70,71,72,73,74,75,76]. These methodologies often integrate instrumental analysis, analytical biology, and materials science to achieve enhanced detection performance. For example, the combination of ICP-MS and CRISPR/Cas12a has greatly improved detection ability (with a range of 0.5 fM–100 pM for miR-21 detection) [67]. An electrochemical biosensor for miRNA with strand displacement amplification (SDA) and tetrahedral DNA nanotags was developed, featuring a detection ability in the range of 100 aM to 10 pM and an LOD calculated to be 75 aM. Although electrochemistry still offers good bio-sample detection capabilities, the preparation of electrodes leads to an increase in the duration of the experimental cycle (approximately 8 to 24 h for the preparation of electrodes). In contrast, fluorescence and colorimetry only take 30 min, and their biological detection capabilities can also reach the 10 fM level, ranging from 0 to 500 fM.

When developing clinical bio-sample detection methods in the future, we should pay attention to the optimization of detection time because the expression levels of some biomarkers in human body fluids are not very low. Excessively pursuing an ultra-low detection limit may lead to overly complex methods and prolonged detection time, thus hindering the widespread application of methods.

### 4.2. The Application of miRNA Detection Utilizing POCT for Disease Diagnosis

Numerous strategies integrating nucleic acid isothermal amplification with electrochemical, spectroscopic, and liquid chromatographic techniques for miRNA-related disease diagnosis in laboratory settings have been developed, but their reliance on bulky instrumentation, complex sample pretreatment, and stringent reaction conditions renders them unsuitable for disease diagnostics in remote regions. Therefore, the integration and intelligent development of portable devices have revolutionized the application of miRNA detection, particularly through the introduction of microfluidic technology and smartphone platforms, which have driven the transformation of miRNA detection toward POCT. This shift has not only enhanced the convenience of miRNA detection but also significantly improved its efficiency and adaptability across diverse applications. Recent advancements in portable devices have demonstrated remarkable potential in revolutionizing miRNA diagnostics, particularly in resource-limited settings and emergency scenarios [77,78,79]. We have summarized research articles on the use of POCT for disease diagnosis in the past three years (2023–2025) [80,81,82,83,84], as shown in Table 4. From our analysis, it is evident that reducing instrument usage shortens detection time, yet the LOQ remains suboptimal. Therefore, in future efforts to develop POCT methods, enhancing detection sensitivity should be prioritized to elevate diagnostic accuracy.

The integration of microfluidics and smartphones into miRNA detection systems offers several advantages. First, optimized amplification protocols and detection algorithms ensure accurate identification of low-abundance miRNAs, even in complex biological samples. Second, automated workflows and intuitive interfaces allow non-specialized personnel to perform tests with minimal training, expanding the accessibility of miRNA diagnostics. Third, these systems’ lower material costs and lack of a requirement for bulky laboratory equipment make them economically viable for widespread adoption. Finally, portable devices can be adapted for various applications, including emergency diagnostics, on-site testing, and decentralized healthcare services.

Wearable biosensors are widely used in POCT. For example, technology enabling the determination of the glucose content of human blood, allowing the continuous monitoring of changes for one week, is available on the market [85]. Wearable devices for detecting miRNA have also been widely studied and applied to disease diagnosis in POCT [86,87,88,89,90]. The detection principle of this wearable technology for detecting miRNA is simpler than that of the nucleic acid isothermal method used in the laboratory. It only requires DNA-RNA hybridization, avoiding complex reaction principles. Meanwhile, the use of more advanced materials and electrochemical technology reduces the detection time to only 10 min, enabling the detection of 1.92 fM target miRNA [87]. Therefore, wearable devices for miRNA detection show great potential, offering advantages such as improved detection performance and shorter disease diagnosis.

In conclusion, the integration of microfluidic technology with smartphone platforms represents a transformative approach to miRNA detection, enabling rapid, cost-effective, and user-friendly diagnostics. These innovations are promising in terms of revolutionizing miRNA diagnostics, ultimately improving patient outcomes through early and accurate disease detection.

### 4.3. The Application of miRNA Detection Utilizing AI Assistance in Disease Diagnosis

Artificial intelligence (AI) refers to computational systems capable of performing tasks that typically require human intelligence, such as problem-solving and data analysis [91]. Machine learning, a subset of artificial intelligence, involves learning from inputs of training data and then identifying similarities and patterns within the data to generate outputs for various purposes, such as data analysis and prediction. Unlike traditional programming, machine learning programs learn autonomously from large quantities of input data, adapt to perform specific tasks, and do not require explicit programming [92]. Advancements in AI can be integrated with biosensors to enhance their performance in disease diagnosis. AI, after being trained on datasets, can swiftly process the data detected by biosensors. This integration has not only transformed conventional diagnostic methods but also accelerated the pace of disease diagnosis. As shown in Table 5, we have summarized studies that employed nucleic acid isothermal amplification methods combined with AI for the auxiliary diagnosis of miRNA-related diseases between 2020 and 2025 [93,94,95,96,97,98,99,100,101].

AI-assisted disease diagnosis saves time and, particularly when combined with deep learning, significantly enhances diagnostic accuracy, demonstrating powerful abilities in processing extensive medical datasets.

## 5. Conclusions

For the past few decades, the field of miRNA detection has primarily relied on traditional methods for analyzing miRNA in biological samples, including Northern blotting, RT-qPCR, and microarray techniques. Given the smallness, high sequence homology, and low abundance of miRNAs, traditional methods for miRNA analysis are hindered by several limitations. Amplification-free methods, such as Northern blotting and microarray technology, exhibit low sensitivity and struggle to quantitatively analyze low-concentration miRNAs in biological samples. Although improved methods offer higher sensitivity, the associated analysis costs are much higher. Consequently, nucleic acid amplification methods are typically employed for the quantitative analysis of miRNAs. Isothermal amplification has emerged as a potent method for miRNA quantitative analysis. In comparison to RT-qPCR, isothermal amplification requires simpler conditions, obviating the need for costly PCR thermal cyclers, and can more rapidly and efficiently detect short RNA sequences without requiring precise control of temperature cycling. Moreover, isothermal amplification techniques overcome PCR’s limitation of being unsuitable for living-cell analysis and can be conducted intracellularly. This review summarizes recent advances in the design and application of isothermal amplification methods. Developing highly sensitive miRNA detection methods based on isothermal amplification bears significant implications for miRNA cellular functions and disease associations. Despite significant technological advancements, miRNA detection is still subject to multiple challenges.

Firstly, for enzyme-free nucleic acid isothermal signal amplification systems, current methods such as CHA and HCR have simple steps. However, they typically require labeled quenching groups and fluorescent tags, increasing detection costs. Furthermore, the design of hairpin primers lacks available software support, posing additional challenges. Additionally, although anti-FITC-HRP and luminol-H_2_O_2_-mediated chemiluminescence detection methods exhibit high detection power, their immunoreactions for recognizing FAM molecules are cumbersome and lack high-throughput detection capabilities.

Moreover, increasing evidence suggests that individual miRNAs are often associated with various diseases, and their expression levels may either increase or decrease during disease progression. Compared to single-miRNA detection, multi-miRNA detection can significantly reduce false-positive rates in disease diagnosis. However, detecting multiple miRNAs requires multiple primers and does not allow simultaneous detection in a single analysis, leading to lower analytical efficiency and higher costs.

Finally, to address these challenges, future research should focus on developing software for CHA and HCR hairpin primer design and synthesizing chemiluminescent reagents that can specifically recognize dsDNA. Such advancements would simplify the signal outputs, enhance detection limits, and reduce the costs of isothermal miRNA detection strategies. Additionally, leveraging chromatographic separation capabilities to construct analytical methods for simultaneous multi-miRNA detection could provide new insights into miRNA biological functions and improve the sensitivity of disease diagnostics. For wearable sensors, the method of detecting miRNA for disease diagnosis does not require a nucleic acid amplification step because it relies on emerging materials with better performance. Therefore, if an enzyme-free nucleic acid signal amplification component is added to wearable sensors, the diagnostic sensitivity will be increased. Overall, overcoming these challenges and exploring innovative approaches will pave the way for more efficient, reliable, and cost-effective miRNA detection technologies.

The revolutionary advancements in miRNA detection technology have not only deepened our understanding of the molecular mechanisms underlying diseases but also demonstrated transformative potential in clinical diagnostics and personalized therapies. From the synergistic innovation of isothermal amplification and CRISPR to the intelligent integration of portable devices, these developments collectively outline a blueprint for future diagnostic technologies characterized by efficiency, precision, and accessibility. As interdisciplinary collaborations continue to deepen, miRNA detection holds the promise of overcoming existing limitations and plays a more central role in early cancer screening, infectious disease monitoring, and chronic disease management. Ultimately, these advancements are aimed at achieving a comprehensive translation from laboratory research to clinical practice, paving the way for impactful and widespread applications in healthcare.

## Figures and Tables

**Table 1:** The diagnostic potential of miRNA derived from multiple different biological samples with respect to cancer.

Types of Biological Fluids	Diagnostic Potential for Cancer	Main Source	Advantages and Limitations	Reference
Serum	Various cancers	Encapsulated in exosomes or bound to proteins	This method avoids degradation of endogenous RNase, and miRNA can be specifically expressed	[9]
		Passive leakage		
		Active secretion	This method requires complex sample pretreatment and biological matrices	
Saliva	Oral squamous cell carcinoma, colorectal cancer, liver cancer, head-and-neck cancer	Salivary glands, gingival crevice fluid, and desquamated oral epithelial cells	Saliva collection is noninvasive and painless	[10,11,12,13,14]
			There is a risk of viral/bacterial contamination, and there are no dedicated miRNA isolation kits	
Urine	Renal tumors, bladder tumors, prostate cancer	Active secretion by various tissues and cells	Such miRNAs yield results directly related to urinary system disease; miRNAs from urine are stable	[15,16,17]
		Secretion of urinary tract cells	There is a low limit of detection and a lack of internal references	

**Table 2:** Multiple-strategy-mediated isothermal amplification methods for miRNA detection.

Analytical Methods	Target	Sample	Required Enzymes	Template/Primers	Temperature (°C)	Time (min)	LOD	Ref
DSNSA (HPLC-FLD)	miR-21, miR-122, miR-155	Human serum	1: DSN enzyme	3	40	200	0.26–0.39 fM	[31]
CHA-HCR-DNAzyme (chemiluminescence)	miR-21	10% human serum	1: DNAzyme	6		120	20.5 pM	[42]
AP-LAMP (fluorescence)	miR-34a	MCF7 cell	2: DNA polymerase and Bst DNA polymerase	6	60–65	90	10 aM	[59]
RCA-LAMP (fluorescence)	miR-let7a	HCT-116 cell	2: T4 RNA ligase and Bst DNA polymerase	4	39 and 65	110	10 aM	[60]
CS-MoS2-CHA (electrochemistry)	miR-21	Human serum	None	4	37	210	16 aM	[61]
DNAzyme-CHA (electrochemistry)	miR-21	Human serum	1: DNAzyme	6	37	285	1 aM	[62]
Gox and MOF nanozyme (ratiometric fluorescence)	miR-21	Human serum	None	4	37	280	0.8 aM	[63]
RCA-CDT (fluorescence)	miR-let7d	A549 cells	1: pi29 DNA polymerase	2	37	90	0.46 fM	[64]
DSN-mismatched CRISPR/Cas12a (fluorescence)	miR-let7a	Human serum	2. Duplex-specific nuclease and Cas12a	4	37 and 50	110	64.17 fM	[65]
DSN-based DNA-modified nanochannels (nanochannel)	miR-21	MCF-7 and HeLa cells	1. Duplex-specific nuclease	1	50	120	1 fM	[66]

**Table 3:** The application of miRNA detection methods utilizing isothermal signal amplification in disease diagnosis.

Analytical Methods	Diseases and Target miRNAs	Sample	Sample Preparation	Determination Time (min)	LOQ	Ref
SDA and CRISPR/Cas12a (spICP-MS)	Breast cancer, miR-21	Human serum and blood	Total RNA extraction	145	0.5 fM	[67]
SDA and SPAAC ligation (electrochemistry)	Non-small-cell lung cancer, miR-21	Human tissue	Total RNA extraction	110	100 aM	[68]
PICA (fluorescence)	Colorectal cancer, miR-21	10% Human serum	Total RNA extraction	30	20 pM	[69]
Au metallene/luminescence nanovesicle (electrochemiluminescence)	Coronary artery calcification, miR-126-3p	Human blood	Total RNA extraction	137	1 fM	[70]
MS-ACPE (fluorescence)	Oral squamous cell carcinoma, miR-31	Human saliva	High-speed centrifuging	440	10 pM	[71]
Dual-mode signal amplification (fluorescence and colorimetric)	Gestational diabetes mellitus, miR-135a	Human plasma	Total RNA extraction	37	0.56 and 8.3 nM	[72]
AIE-split G-quadruplex (fluorescence)	Acute kidney injury, miR-21	10% Human urine	Centrifugation	30	10.36 fM	[73]
AuNP@LH (fluorescence)	Systemic lupus erythematosus, miR-146a, miR-29c, and miR-150	Human urine	Centrifugal Filter (100 kDa)	110	50 pM	[74]
Mango II arrays (fluorescence)	Lung and liver cancer, miR-21	50% Human serum	Centrifugation and incubated at 95 °C	180	10 fM	[75]
Plasmonic nanocavity-modulated ECL (electrochemiluminescence)	Gastric cancer, exosomal miR-223-3p	Human ascites	Centrifugation and exosome Kit	Not mentioned	1 fM	[76]

**Table 4:** The application of miRNA detection methods utilizing POCT in disease diagnosis.

Analytical Methods	Diseases and Target miRNAs	Sample	Sample Preparation Method	Determination Time (min)	LOQ	Ref
Microfluidics and smartphone (DSNSA)	Type 2 diabetes, miR-21	Human serum	Total RNA extraction	150	1 pM	[80]
Smartphone (EXPAR)	Bladder cancer, prostate cancer, miR-223, and miR-155	Human urine	Total RNA extraction	22.5	1 pM	[81]
Smartphone (SDA and HCR)	Lung cancer and miR-21	1% Human serum	Not mentioned	60	0.1 fM	[82]
Lateral flow assay and smartphone (RCA with CRISPR–Cas12a)	Pancreatic cancer, miR-21, miR-451a, and miR-1246	Human plasma	Total RNA extraction	20 to 180	20, 10, and 50 fM	[83]
Lateral flow assay (CHA and AuNPs)	Liver cancer and miR-223	Human serum	Not mentioned	40	1 pM	[84]

**Table 5:** The application of miRNA detection methods utilizing AI assistance in disease diagnosis.

Analytical Methods	Diseases and Target miRNAs	Sample	Diagnosis Accuracy	Diagnosis Time (min)	AI Types	Ref
Localized CHA (LCHA) and PMSD	Non-small-cell lung cancer, miR-182, miR-21, miR-148, let-7b, miR-143, and miR-30a	Human tissues	92.86%	150 (sample to diagnosis)	Machine learning	[93]
CRISPR/Cas13a	Colorectal cancer, miR-16–2, miR-375, miR-378a, and miR-7	Human fecal	97.4%	100	Machine learning	[94]
Target-triggered catalytic hairpin assembly	Coronary artery disease, miR-21, miR-155, miR-1	50% Human serum	87.5%	90	Machine learning	[95]
Dumbbell probe-mediated CRISPR/Cas13a with nicking-induced DNA cascade reaction (DP-bridged Cas13a/NDCR)	Colorectal cancer, miR-17, miR-21, miR-182, and miR-223	Human blood plasma	100%	120	Machine learning	[96]
Size-coded hydrogel microbeads	Lung cancer, miR-21, miR-205, and miR-375	Human serum	80% (lung cancer sub-type prediction)	120	Machine learning	[97]
Nanoflare probe, CHA amplification, and total internal reflection fluorescence (TIRF)	Lung cancer, breast cancers, colon cancers, and cervical cancers, miR-21, miR-122, mir-375	Human plasma	100%	120	Deep learning	[98]
DNA-encoded plasmonic-bubble-driven SERS	Liver cancer, miR-21, and miR-155	Human blood	83.3%	Not mentioned	Decision-tree-based classifier algorithms	[99]
Nanosatellites (magnetic beads (MBs) @ NaLnF_4_) and CHA amplification in combination with ICP-MS	Breast cancer, lung cancer, stomach cancer, colon cancer, and cervical cancer, miR-200b, miR-21, miR-151, miR-155, miR-139, let-7b, miR-191, miR-214, let-7f, and miR-30e	Human plasma	100%	90	Linear discriminant analysis (LDA)	[100]
Nanoneedle-based discrete analysis	Acute myeloid leukemia (AML), miR-155, miR-21, miR-125b, miR-99a, miR-223, miR-29a, miR-126, miR-181a, and miR-196b	Living AML cells	90%	120	Machine learning	[101]

## Data Availability

No new data were produced in this manuscript.

## Abbreviations

The following abbreviations are used in this manuscript:

MiRNA	MicroRNA
MS-ACPE	Magnetic-separation-assisted auto-cyclic primer extension
RT-qPCR	Reverse transcription quantitative PCR
ncRNA	Non-coding RNA
PTC	Papillary thyroid carcinoma
FNAB	Fine needle aspiration biopsy
POCT	Point-of-care testing
PICA	Product-induced catalysis amplification
RCA	Rolling circle amplification
DSNSA	Duplex-specific nuclease signal amplification
CHA	Catalytic hairpin assembly
SDA	Strand-displacement amplification
SPAAC	Strain-promoted azide–alkyne cycloaddition
HCR	Hybridization chain reaction
LAMP	Loop-mediated isothermal amplification
LOQ	Limit of quantitation
EXPAR	Exponential amplification reaction
ECL	Electrochemiluminescence
dNTPs	Deoxyribonucleoside triphosphates
AuNPs	Gold nanoparticles
AuNP@LH	Target-triggered locked hairpin DNA-functionalized Au nanoprobes
AIE	Aggregation-induced emission
FRET	Fluorescence resonance energy transfer
HPCL-FLD	High-performance liquid chromatography coupled with a fluorescence detector
ICP-MS	Inductively coupled plasma–mass spectrometry
HP	Hairpin primer
dsDNA	Double-stranded DNA
FIP	Forward inner primer
BIP	Backward inner primer
